# Editorial Notes

**Published:** 1904-06

**Authors:** 


### Editorial Notes.

**Bristol Royal Infirmary.**

**President and Treasurer.**

THE resignation of Sir Charles Daniel Cave, Bart., of an office which he has held for the long period of twenty-four years calls for some recognition of his services, which have been many and great. Appointed in 1880, his tenure of office has been two years longer than that of his father, Mr. Daniel Cave, who resigned in 1844, and nearly sixteen years longer than the average (8.8 years) of the sixteen Presidents, who commenced with John Elbridge in 1735. His activity as President is shown by the long list of life governors and donors of upwards of thirty guineas in the year 1881, when a special effort was made to pay off a debt which had been accumulating for some years. Since that time no special appeal has been made, but numerous extensions and improvements have necessitated a very large and increasing expenditure, which exceeds the ordinary income by the sum of four thousand pounds. At a Meeting of the Board held on Friday, May 5th, the very Rev. the Dean of Bristol moved: "That this Board of Governors accept the resignation of Sir Charles D. Cave with profound regret, and unanimously desire to place on record their appreciation of the signal services which he has rendered as President and Treasurer for nearly twenty-five years, and of the unfailing courtesy and patience which have endeared him to all his colleagues. They trust that he may be spared for many years to watch with interest the institution over whose destinies he has so long presided." The Board then proceeded to elect a new president, and Dean Pigou said "they all felt that the president of a great institution like that, with its traditions and importance in the place, should be pre-eminently a citizen of Bristol who knew its wants, and was in touch with the people; should be a man of great tact and knowledge, of business habits, and with considerable influence among his fellow-men. The name he had to submit for their consideration was a household word in Bristol. Mr. George White had shown devotion to this city, and had identified himself already with many philanthropic matters. He had not shrank from the position of president for reasons which would make some men hesitate and think twice, but felt rather that he had a special call to undertake the office. He congratulated, not only the Infirmary, but Bristol generally, upon the prospect of having as president a man who would fill the conditions he had ventured to mention as fitting to the office. Mr. George White would certainly have the confidence of the city, and would bring fresh blood and fresh experience into their midst." The following letter from Mr. George White was then read by the Secretary, and is such that it should be placed on record as a notable example of public spirit and self-sacrifice: "I have given much thought to the suggestion so kindly conveyed to me that I should accept the presidency of the Bristol Royal Infirmary, in the place of Sir Charles Cave, who, I understand, is desirious of retiring. In view of the present position of the finances of the institution, I appreciate the fact that the office would entail considerable anxiety and responsibility, but I am also free to confess that it is just these difficulties of the committee which seem to me to constitute an irresistible claim upon the services of any Bristolian, and my duty, therefore, is obvious, especially so at this critical juncture in the life of an institution which is of such inestimable value to thousands of our less fortunate fellow-citizens. There is clearly a pressing necessity for at once raising a sum of £20,000, but with the aid of such an experienced body as the committee of the Infirmary this task should not be insurmountable. When we remember, too, the generosity with which Bristol always responds to any deserving appeal, and, moreover, that the claims of this institution are pre-eminent amongst those of the charities of our city, we can look forward with every confidence to complete success in the great efforts which will have to be put forth. It is only proper that I should remind the committee that I am entirely without experience in such work, but, if there is still a general desire that I should accept the office, I may say that I shall feel honoured by their selection, and shall look forward with pleasure to an association with those who have for so many years devoted themselves to the interests of this noble charity. Believe me to be, very sincerely yours, George White". All friends of the Infirmary will wish the New President a long career of satisfactory progress in his new office, and will congratulate the Institution in having at its head one whose well-known knowledge of finance and whose philanthropic spirit are likely to be of incalculable value. Whatever course may ultimately be adopted as regards the outstanding debt to the Treasurer, amounting at the close of last year to the large sum of £15,548, it should be remembered that the financial stability of the Institution is to some extent secured by the presence of what may be called a reserve fund of capital amounting to £133,261. It would not be honourable that this should be ruthlessly utilised by the present generation for the purpose of paying its recent debts, so that doubtless some effort must be made in the near future to increase the annual income and to pay off the accumulated liability of many past years. But, further, it is much to be desired that some steps shall be taken to curtail the expenditure of an institution whose income is annually far less than the former. This cannot be done by closing wards which are constantly full of deserving cases, and the general principle of municipalisation of hospitals has received (and, we think, rightly so) very little favour in this country. The question of hospital abuse is raised from time to time, and some persons are inclined to credit the authorities of our medical institutions with laxity in their standard as to the qualification for hospital treatment. The motto of the Infirmary, "Charity Universal," almost forbids too close a scrutiny of the financial position of the applicants for relief, but an investigation by the Charity Organisation Committee some years ago tends to show that here as elsewhere there is some misuse of the time and gratuitous service of the medical staff by those who could well afford to pay a moderate fee, and that some of the funds of the institution are in this way misapplied,

**Medical Defence.** THE proposal to combine the various societies under the all-embracing wing of the British Medical Association seems to have led to much discussion and not a little disagreement. The Committee appointed by the Association has drawn up a draft scheme, which is being submitted to the consideration of the local divisions. It remains to be proved whether that consolidation which is proposed can in any way be effected. It is felt that the London and Counties Medical Protection Society and the Medical Defence Union with other societies engaged in medical defence have done good work for many years, and it seems a little unreasonable to ask that they should cease to exist. It is also felt that the work may not be done so efficiently by a sub-committee of the Council of the British Medical Association as it has already been done by those who have undertaken it *con amore* and have carried it on so efficiently. The Bristol division has given its vote against the scheme as proposed, but it remains open to the individual members of the Association to choose for themselves which scheme of medical defence they consider to be the most worthy of their confidence.

**Dr. Maclagan and his great work.** THE death of Thomas John Maclagan, M.D., has been the subject of an article in the *Nineteenth Century* by Sir William Broadbent, Bart., K.C.V.O., M.D., F.R.S., under the above heading. The utility of salicin and the salicylates is now so universally admitted that the origin of the discovery and the discoverer are apt to be forgotten; but this article calls attention to the miasmatic theory of rheumatism, the presence of a specific poison and its antidote. It was insisted on by Maclagan that "to attain the object the remedy must be administered in full doses . . . that to give salicin in iron-tonic fashion, ten grains three times a day or every four hours, was to bring it into discredit and to court failure: to fulfil the required indication and kill the organism twenty or thirty grains should be given every hou for six doses in two succeeding days." Recent investigations on rheumatism have more than confirmed Maclagan's views, and his name should be enrolled as one of the great benefactors of the human race.

**The State Registration of Nurses.**

Opinions seem to be much divided on this question. Miss Eva C. E. Lückes, matron of the London Hospital, than whom there is no one who can speak with more authority, in the *Nineteenth Century* for May, sums up the arguments against the proposed registration in a very able article, showing that recognition by the State will not be a panacea for all the real and imaginary grievances against nurses. The article concludes as follows:—"Many will be ready to defend the freedom of the best-trained nurses, and to do all that is possible to save them from the fallacious 'protection' with which they are threatened. If the unsuitable could be annihilated by registration, there would be something to say in its favour; but no one can claim that this would be achieved." Many have already done this. The letter of the chairman of the London Hospital[1] makes the statement that "the matrons of almost all the leading training schools for nurses are opposed to registration, and will not serve on any such council."

**Ministry of Public Health.**

The proposal to institute some reform for the removal of flaws in our sanitary organisation has been favourably received by many sanitary associations. The British Medical Association so far sympathises with the need for a stronger State Department of Health that it is promoting a Bill in Parliament for the remodelling of the Local Government Board, making the President of the Board a Secretary of State. Dr. F. Bushnell, of Plymouth, calls our attention to the fact that this subject is to be discussed at an early meeting of the Royal Institute of Public Health, and he suggests that the following questions might be taken as subjects for discussion:—(1) The duties of the proposed Ministry in contrast with those of the Local Government officer. (2) The co-ordinate action of the latter and other offices in public health matters with the

[1] *Brit. M. J.*, 1904, i. 920.

proposed Ministry. (3) Its cost and its advantages. (4) The international aspect of the State towards hygiene and medicine. There seems to be no doubt that the subject is one of such importance that it should be made an official one in all international and other sanitary and medical congresses. We notice in this connection that an institute of hygiene is being formed for the purpose of promoting the advancement of personal hygiene, with the following objects:—To institute classes, lectures and demonstrations; to form a library of works on personal and domestic hygiene; to publish a journal devoted to personal and domestic hygiene; to establish a permanent exposition of food products, beverages, articles of clothing and appliances representative of home and foreign progress in each of these departments; and to have a recognised centre of information on all such matters as pertain to personal and domestic hygiene.

**Winsley Sanatorium for Consumptives.**

THE annual meeting of the Gloucester, Wilts and Somerset Branch of the N.A.P.C., held on May 13th at the Bristol Council House, was opened by the Lord Mayor of Bristol, Sir John Dickson Poynder, Bart., M.P., presiding. In presenting the annual report, Dr. Lionel Weatherly pointed out that no less than thirty-five beds were being built and maintained by corporations, rural district councils, working men's associations, private firms and private individuals. They expected that the sanatorium would be ready for opening in November. We regret that a sum of about £6,000 is still needed to start free from debt the sanatorium for sixty patients.

**Long Fox Lecture.**

THE first of the annual lectures in memory of the late Dr. Edward Long Fox will be delivered in November next by Dr. John Beddoe, F.R.S. We congratulate the appointing board on their fortunate selection, and on being able to secure the services of one who while being a former colleague of the late Dr. Long

Fox at the Bristol Royal Infirmary has made his name illustrious amongst British scientists, and we have no doubt that the coming lecture will be of lasting interest and value. The precise date and time will be announced in a later issue.

---

### Notes on Preparations for the Sick.

